# The Plague of Thebes, a Historical Epidemic in Sophocles’ Oedipus Rex

**DOI:** 10.3201/eid1801.AD1801

**Published:** 2012-01

**Authors:** Antonis A. Kousoulis, Konstantinos P. Economopoulos, Effie Poulakou-Rebelakou, George Androutsos, Sotirios Tsiodras

**Affiliations:** University of Athens Medical School, Athens, Greece (A.A. Kousoulis, K.P. Economopoulos, E. Poulakou-Rebelakou, G. Androutsos, S. Tsiodras);; Society of Junior Doctors, Athens (A.A. Kousoulis, K.P. Economopoulos)

**Keywords:** bacteria, zoonoses, plague of Thebes, Sophocles, drama, Oedipus Rex, epidemic, Brucella abortus, brucellosis, plague of Athens, Greece, literature, history of medicine

## Abstract

Sophocles, one of the most noted playwrights of the ancient world, wrote the tragedy Oedipus Rex in the first half of the decade 430–420 bc. A lethal plague is described in this drama. We adopted a critical approach to Oedipus Rex in analyzing the literary description of the disease, unraveling its clinical features, and defining a possible underlying cause. Our goals were to clarify whether the plague described in Oedipus Rex reflects an actual historical event; to compare it with the plague of Athens, which was described by Thucydides as occurring around the same time Sophocles wrote; and to propose a likely causative pathogen. A critical reading of Oedipus Rex and a comparison with Thucydides’ history, as well as a systematic review of historical data, strongly suggests that this epidemic was an actual event, possibly caused by *Brucella abortus*.

## Abstract 2

*Brucella abortus* may have been the etiologic agent.

”Wailing on the altar stair, wives and grandams rend the air, long-drawn moans and piercing cries blent with prayers and litanies”—Sophocles, Oedipus Rex, lines 184–186

Sophocles is one of the most noted playwrights of the ancient world and, along with Aeschylus and Euripides, belongs to the trinity of the Attic tragedians who flourished during the golden century of Pericles in Athens (Figure 1). Sophocles lived between 496 and 406 bc; although he seems to have written 123 plays, only 7 have survived in a complete form (*1*). He lived his entire life in Athens and introduced many innovations in the dramatic arts (*1*).

**Figure 1:**
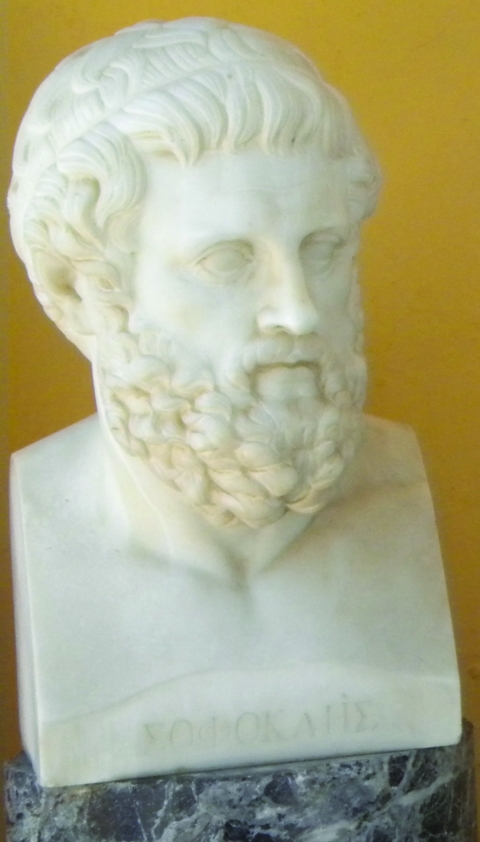
Bust of Sophocles in the Colonnade of the Muses in the Achilleion, Corfu, Greece, July 2011. Photo courtesy Antonis A. Kousoulis.

The writing of the tragedy Oedipus the King (original Greek title Οιδίπους τύραννος, most commonly known as Oedipus Rex) is placed in the first half of the decade 430–420 bc. The play has been labeled an analytical tragedy, meaning that the crucial events which dominate the play have happened in the past (*2**,**3*).

Oedipus Rex, apart from the undeniable literary and historic value, also presents significant medical interest because the play mentions a plague, an epidemic, which was devastating Thebes, the town of Oedipus’ hegemony. Several sections, primarily in the first third of the play, refer to the aforementioned plague; the epidemic, however, is not the primary topic of the tragedy. The epidemic, in fact, is mostly a matter that serves the theatrical economy by forming a background for the evolution of the plot.

Given the potential medical interest of Oedipus Rex, we decided to adopt a critical perspective by analyzing the literary descriptions of the plague, unraveling its clinical features, defining the underlying cause, and discussing possible therapeutic options. The ultimate goals of our study were to clarify whether the plague described in Oedipus Rex could reflect an actual historical event, compare it with the plague of Athens, which was described by the historian Thucydides as occurring not long before the time that Sophocles’ work appeared (*4*), and propose the most likely causative pathogen.

## An Epidemic in Oedipus Rex

In the first scene of the play, Sophocles presents the basic social and historical axes around which he will unfold the plot. The devastating plague that dominates Thebes is presented to the audience through the dialogue between Oedipus and the Priest (lines 1–67) (*2**,**3*). The king has already taken some action to deal with this harm by sending his brother-in-law, Creon, to the oracle at Delphi to ask for a salvation plan (lines 68–72). The oracle announces that the plague is a result of religious pollution and that the god Apollo requests that the people of Thebes exile the previously unknown “miasma” (a word of Greek origin with a sense of moral noxious pollution) away from the town (lines 96–98) (*2**,**3*). Oedipus asks the citizens to stop praying and focus on finding the cure (lines 142–146) (*2**,**3*). In lines 167–215 the Chorus stays on stage to summarize the situation and beg for salvation (*2**,**3*).

Searching for the miasma, Oedipus summons the blind prophet Tiresias to reveal who is responsible for this evil (lines 300–313) (*2**,**3*). At the moment that Tiresias reveals to Oedipus that the king himself is the cause of the plague (lines 350–353), the epidemic becomes a secondary issue, and, as a result, there are only occasional references to the plague during the remainder of the play (lines 665–666, 685–686, 1380–1383, 1424–1428) (*2**,**3*).

**Figure 2:**
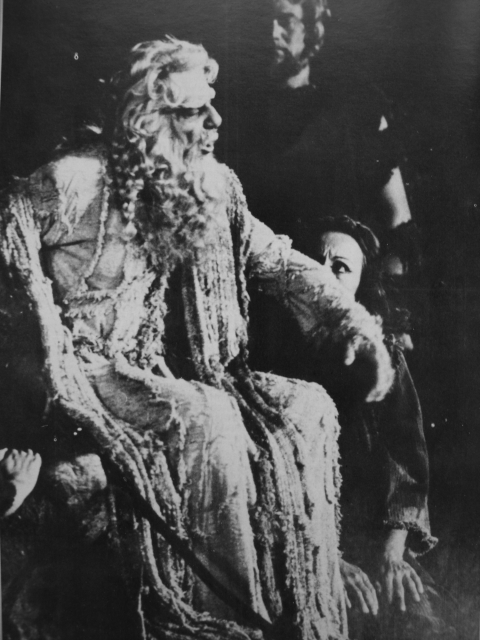
Scene from a National Theatre of Greece production of Oedipus Rex at the Odeon of Herodus Atticus, Athens, Greece January 1995. Photo courtesy Effie Poulakou-Rebelakou.

Therefore, although the first part of the play is rife with references to the plague and its consequences, in the second part there are only sporadic referrals to the epidemic. The fate of Oedipus emerges as a truly tragic one, not so much because he caused the plague, but because of the character’s own personal tragedy (Figure 2).

### A Medical Critical Approach to Oedipus Rex

From the start of the drama, the plague in Thebes is a serious matter, as in line 23 where it is referred to as “weltering surge of blood” (φοινίου σάλου). In line 28 the word plague (λοιμός) appears for the first time, with the Greek word for disease (νόσος) being used in lines 150, 217, and 303 (*2**,**3*).

Sophocles describes the main characteristics of the epidemic through sporadic sentences. Early in the play it is clarified that the disease is a cattle zoonosis of high mortality rate (“a blight upon the grazing flocks and herds,” line 26, with the herds being cattle,) (*2**,**3*). The lethality of this epidemic is particularly terrifying for the protagonists of the play, and the disease’s severity is evinced by the first sentences of the tragedy (“reek of incense everywhere,” line 4). Oedipus fears mass destruction of the city of Thebes (“with the god's good help success is sure; 'tis ruin if we fail,” line 146), while the words “weltering surge of blood” (line 24), “fiery plague” (line 166), “the land is sore distressed” (line 685), and “wailing on the altar stair, wives and grandams rend the air, long-drawn moans and piercing cries blent with prayers and litanies” (lines 184–186) (*2**,**3*) all illustrate vividly the severity of the situation.

The references to the decline of land and fields could be an example of poetic exaggeration or a suggestion that the fruits or ears may participate in the transmission route of the plague (“a blight is on our harvest in the ear,” line 25) (*2**,**3*). Regarding the specific clinical features of the disease, it is clear that the causative pathogen leads to miscarriages or stillbirths (“a blight on wives in travail,” lines 26–27, meaning women give birth to dead babies) (*2**,**3*). The plague’s effects are also pointed out by the Chorus: “earth her gracious fruits denies” and “women wail in barren throes” (lines 151, 215) (*2**,**3*).

Lines 179–181 turn out to be of high interest: “wasted thus by death on death all our city perishes; corpses spread infection round” (*2**,**3*). A word with a meaning of something that brings death is used in the original Greek (θαναταφόρα) to refer to the plague, which suggests that at the time of Sophocles his fellow Greeks were aware of the threat posed by infectious disease. The knowledge of the existence of a highly contagious and fatal disease is phrased clearly in these rhymes, strongly suggesting that Thebans were aware of the oncoming—most possibly from the adjacent city of Athens—danger (*2**–**4*). This hypothesis regarding the source of the disease seems the most reasonable in medical terms, contrary to the philological approach, which declares that the epidemic derived from the gods.

In addition, the Chorus provides us with a major social aspect, as they put the blame on god of war, Ares (“Ares whose hot breath I feel, though without targe or steel he stalks, whose voice is as the battle shout,” lines 190–191) (*2**,**3*). It is not quite clear why Ares is being called responsible for this plague, since there is no other such reference in the play. In fact, it is noteworthy that there is no other historic or poetic reference that links Ares to the spreading of a disease (*4*). However, Thucydides’ correlation of the plague of Athens with the Peloponnesian War (431–404 bc) (*5*) gives us the opportunity to state that Sophocles connects this epidemic of Thebes with the plague of Athens and attempts to point out the disastrous effects wars always have.

Regarding the play’s approach to treatment of the disease, reading through the drama we once again come across with the theocratic perceptions of ancient Greece. The citizens have become suppliants to the monuments of the gods, asking for mercy (“Why sit ye here as suppliants, in your hands branches of olive filleted with wool?,” lines 2–3; “the common folk, with wreathed boughs crowd our two market-places, or before both shrines of Pallas congregate, or where Ismenus gives his oracles by fire,” lines 19–21) (*2**,**3*). Consequently, a solution for the situation is requested from the oracle at Delphi (lines 68–72), while the Chorus plead for Athena, Zeus, Artemis, and Apollo to save the town from the disaster (lines 160–165) (*2**,**3*). The aforementioned aspects strongly support the notion that the disease was incurable at this time.

### Possible Pathogens Responsible for the Plague in Thebes

The pathogen of the plague described in Oedipus Rex reflects the complexity of every historically emerging zoonosis. Any proposed pathogen should be a highly contagious, zoonotic disease of cattle that causes stillbirth, miscarriages, and infertility, is characterized by high mortality rates, and has the potential to have caused an epidemic in the 5th century bc. The characteristics of pathogens that might be responsible for the plague on the basis of Sophocles’ descriptions in Oedipus Rex are summarized in the Technical Appendix.

After a close inspection of the characteristics, the pathogens that include most (5 of 7) of the features described by Sophocles in Oedipus Rex are *Leishmania* spp., *Leptospira* spp., *Brucella abortus*, *Orthopoxviridae*, and *Francisella tularensis*. Among the diseases caused by these pathogens that can affect humans are the following: 1) tularemia, which is a disease mainly transmitted through rabbits; 2) smallpox, which is not a cattle zoonosis; 3) l**eishmaniasis, which** is not a highly contagious disease; and 4) leptospirosis, which has been associated with epidemics after rainfall and flooding in relation to rodent infestation. Thus, the most probable cause of the plague in Thebes is *B. abortus*. Brucellosis is a highly contagious zoonosis caused by ingestion of unsterilized milk or meat from infected cows or close contact with their secretions. Τhe mortality rate for untreated brucellosis is difficult to determine from the literature of the preantibiotic era (*6*); nevertheless, an 80% rate has been reported in situations of comorbidity with endocarditis (*7*). Epidemics, stillbirths, and miscarriages caused by *B. abortus* have been reported since the time of Hippocrates, which is when this disease was initially described.

However, taking into account that in modern times brucellosis in humans is a severe granulomatous disease characterized by extremely rare direct transmission from person-to-person, insidious onset in sporadic cases (mainly among veterinarians), and low mortality rates, it may be difficult for 21st century physicians and veterinarians to accept *B. abortus* as the causative agent of the plague of Thebes. Alternatively, the plague of Thebes could be a composite of >2 causative agents, as it has been suggested for the contemporary plague of Athens (*6**,**7*). In this case scenario, we could assume that cattle in Thebes may have been having brucellosis, leptospirosis, or listeriosis, while humans could have been affected by a different pathogen such as *Salmonella enterica* serovar Typhi (*8**,**9*). It should be noted that exploring the diseases of history requires examining the social, economic, and demographic aspects of each era because this is the only way to better understand how diseases work over centuries (*10*). Finally, we cannot reject the possibility of dealing with a *Brucella* strain that has evolved to become less deadly than a more lethal ancestor (*6*).

### Thucydides’ Plague of Athens and Sophocles’ Epidemic

The plague that is described in Oedipus Rex could possibly be related to the plague that struck Athens in 430–429 bc (*11*), the primary source for which is the papers of historian Thucydides (where he refers to an epidemic that has been named the plague of Athens) (*5*). The following 5 points support this correlation.

#### Proximate Eras

The first writing of Oedipus Rex most probably took place during the time of the plague of Athens. Sophocles’ epidemic seems to have enough strength to appear as a historical base on which the theatrical economy of the play is evolving (*12*). The opening of the drama, with the city of Thebes in the midst of plague has often been, historically, taken as a reference to the plague that devastated Athens in the opening years of the Peloponnesian War and has been used to assist in the dating of this play (*13*).

#### Similar Descriptions by Thucydides and Sophocles

Thucydides and Sophocles use similar terms when describing attempts to deal with the epidemic. In the historical case (Athens) and the dramatic case (Thebes), the populace turned to the temples looking for a divine solution to the disaster.

#### Correlation with Recent Warfare

As mentioned above, in Sophocles’ drama, god of war Ares gets the blame for the plague (lines 190–191). The particularity of this reference (*4*), it seems that Sophocles correlates the epidemic that strikes Thebes with the plague of Athens, which, according to Thucydides, came about as a result of the Peloponnesian War (*5*).

#### Similarities Regarding the Nature of the Diseases

It is difficult to compare a historical record to a poetic drama, but, keeping that in mind, both Sophocles and Thucydides refer to animal illness and death (*2**,**8*). In addition, the realistic descriptions of the historian and the nightmarish lyrical rhymes of the poet, talk about a disease with a high mortality rate (*2**,**10*). As for the clinical features, although Thucydides does not mention the pregnancy or labor pains as described in Sophocles’ text, he does refer to abdominal and vulvovaginal symptoms (*5*).

#### Common Assumptions about the Possible Causative Pathogen

Historical medical literature has suggested many infectious diseases over time, but few have lasted as the most probable. These diseases mainly include typhoid fever, epidemic typhus, smallpox, plague, measles, and influenza, all of which could be initial candidates for the plague in Oedipus Rex and have been taken into account in this study (Technical Appendix) (*9**,**10*).

Although the above points are of great relevance, they lack the possibility of historical verification and are mainly based on the comparative and critical assessment of Sophocles’ and Thucydides’ work. Historical certainty can be added by studying the alliances and the warfare involving Thebes during the era of Sophocles (e.g., Boeotian prefecture, Athenian dynasty, Spartan alliance, Persian wars) (*14*). Bearing in mind the aforementioned observations and the fact no other epidemics were reported in the eastern Mediterranean during the 5th century bc, we posit that the plague described by Sophocles in the tragedy Oedipus Rex has an actual basis in the plague of Athens described by Thucydides in his histories.

## Discussion

A severe plague is described in Sophocles’ drama Oedipus Rex. According to the World Health Organization, an epidemic is defined as a disease outbreak and, therefore, the occurrence of cases of disease in excess of what would normally be expected in a defined community, geographic area, or season (*15*). Thus, according to the tragedy’s rhymes and with respect to literary talk, this plague should be treated as an epidemic.

It would not be irrational to trust the historical credibility of a literary text. Literature usually reflects the echo of the past. A somewhat similar example is that of archeologist Heinrich Schlieman; before Schlieman, the writings of Homer had been considered a collection of mythological poems. However, with his excavations in Troy, which used the Iliad as a guide, Schlieman provided a perfect example of how literary work can have a factual base (*16*).

Moreover, we could not overlook that Sophocles is the most realist of the Greek tragedians (*1*), and ancient tragedies were often placed into a historical frame strongly influenced by major contemporary events (*13*). Finally, although many of the features of the plot and passages have been interpreted as historical allusions, the plague seems to be recognized as the most critical element that reflects a historical event, with enough strength and clarity to be used even for the dating of the tragedy (*12*).

## Conclusions

The critical reading of Oedipus Rex, its comparison with Thucydides’ history, as well as the systematic review of the existing historical data, lead us to strongly suggest that this epidemic, for which the name Plague of Thebes may be used, was an actual historical fact, likely caused by *B. abortus*. With the deadly plague, which struck one of the most historic Greek cities, on the one hand and the tragic fate of a character who has become among the most recognizable in world theater on the other, Sophocles masterminded a dramatic frame and offered a lyrical, literary description of a lethal disease. As the protagonist approached his tragic catharsis, the moral order much desired by the ancient Greeks was restored with the end of the epidemic.

## Supplementary Material

Technical Appendix Characteristics of pathogens possibly responsible for the plague of Thebes described by Sophocles in Oedipus Rex
