# Supplementary material for: The Plague of Thebes, a Historical Epidemic in Sophocles’ Oedipus Rex
Source: Emerg Infect Dis. 2012 Jan;18(1):153–7. doi: 10.3201/eid1801.AD1801 (PMC3310127; doi:10.3201/eid1801.AD1801)
Supplement: Technical Appendix — Characteristics of pathogens possibly responsible for the plague of Thebes described by Sophocles in Oedipus Rex [file AD-1801-Techapp_9p.pdf]

Article DOI: http://dx.doi.org/10.3201/eid1801.AD1801

The Plague of Thebes, a Historical Epidemic in Sophocles' Oedipus Rex

Technical Appendix

Table. Characteristics of pathogens possibly responsible for the plague of Thebes described by Sophocles in Oedipus Rex*

Pathogen	Disease	Zoonosis in cattle	Stillbirth/ miscarriage	Infertility	Highest reported acute mortality rate	Highly contagious (droplets/aerosol transmission)	Epidemics have been described	Described as early as 5th century BC
Yersinia pestis	Plague	No (reported mainly in rodents, cats, camels) (*1*,*2*)	NA	NA	100% (*1*,*2*)	Yes (*2*)	Yes (*1*,*2*)	Probably yes (1350 BC) (*3*)
Leishmania spp.	Leishmaniasis	Yes (*4*)	Yes (≈10.5% for cutaneous and visceral) (*5*)	NA	70% (*6*)	No (sandfly bite) (*7*)	Yes (*7*)	Probably yes (7th century or possibly as easily as 2500 to 1500 BC) (*8*)
Leptospira spp.	Leptospirosis	Yes (*9*)	Yes (in acute infection) (*9*)	NA (reported in animals)	50–70% (*10*)	No (rarely reported direct transmission between humans) (*9*)	Yes, usually in association with rainfalls, flooding and rodent infestation (*9*)	Probably yes (recognized as an occupational hazard of rice harvesting in ancient China) (*9*)
Lyssavirus spp.	Rabies	Yes (*11*)	NA	NA	100% (*11*)	No (*11*)	Yes (*12*)	Yes (Eshuma Code of Babylon circa 2000 BC) (*11*)
Hepatitis E Virus	Hepatitis E (*13*)	Yes (*13*)	Yes (*13*)	NA	4% (*13*) (73% in pregnancy) (*14*)	No (*13*,*14*)	Yes (*13*,*14*)	No (earliest outbreak attributed to HEV was in Delhi, 1955) (*13*,*15*)
Coronaviridae	Common cold, SARS	Yes (*16*)	NA	NA	15% (55% in persons >60 years of age) (*17*)	Yes (*17*)	Yes (*16*,*17*)	No (first characterized in the 1960s) (*18*)
Influenzavirus A	Influenza	Yes (*19*)	Yes (*20*)	No (*21*)	5–10% (*22*) (may be higher) (*23*)	Yes (*24*)	Yes (*22*,*23*)	Yes (*25*)
West Nile virus	West Nile virus infection	No (*26*,*27*)	No (*26*,*27*)	NA	10% (*26*)	No (*26*–*28*)	Yes (*26*,*27*)	Probably not (molecular dating shows low possibility of human infection before 1000 years ago) (*29*)
DEN 1-4 *Flaviviruses*	Dengue and dengue hemorrhagic fever	Not the same virus in humans and cattle (*30*,*31*)	Not in a high rate (*32*)	NA	47% (*33*)	No (*30*)	Yes (*30*)	No (earliest record of Dengue symptoms found in China, during Chin dynasty, 265-420 A.D.) (*30*)

Pathogen	Disease	Zoonosis in cattle	Stillbirth/ miscarriage	Infertility	Highest reported acute mortality rate	Highly contagious (droplets/aerosol transmission)	Epidemics have been described	Described as early as 5th century BC
Brucella abortus	Brucellosis	Yes (*34*)	Yes (*35*)	No (*34*) (only in natural host) (*36*)	80% in cases in which endocarditis was concurrently present (*37*)	Yes (*38*)	Yes (*39*)	Probably yes (known since the time of Hippocrates) (*40*)
Listeria monocytogenes	Listeriosis	Yes (*41*)	Yes (*42*)	No (*43*)	63% (*42*)	No (*42*)	Yes (*42*)	No (early 20th century) (*44*)
Orthopoxviridae	Smallpox	No (*45*)	Yes (*46*)	Yes (*47*)	30% (*48*)	Yes (*48*)	Yes (*48*)	Yes (observations from 1100-1580 BC, even earlier phylogenic origin) (*49*)
Measles virus	Measles	Yes (not reported in cows) (*50*)	Yes (*51*)	No (*52*)	15% (after encephalitis) (*52*)	Yes (*52*)	Yes (*53*)	No (not described BC, and recent phylogenic analysis showed its origin in 11th–12th century AD) (*54*)
Salmonella typhi	Typhoid fever	No (*55*)	Yes (*56*)	NA	30% (when left untreated) (*57*)	No (*58*)	Yes (*59*)	Yes (*60*)
Francisella tularensis	Tularemia	Yes (mainly on rabbits, ticks, deerflies, squirrels) (*61*)	No (reported in sheep) (*62*)	NA	60% (*63*)	Yes (*63*)	Yes (*63*)	Probably yes (earliest outbreak credited, in 1715 BC Canaan) (*64*)
Yersinia enterocolitica	Gastroenteritis	No (mainly in pigs, dogs, cats, sheep and wild rodent strains) (*65*)	No (reported in sows, cattle) (*66*)	NA	50% (after septicemia) (*67*)	No (*65*)	Yes (*68*)	Probably not (Yersinia spp. is an ancient pathogen (*3*), first recognized reference to *Y. enterocolitica* in the USA in 1934 (*69*))
Rickettsia prowazekii	Epidemic typhus	No (mainly in flying squirrels) (*70*)	NA	NA	60% (*70*)	Yes (*70*)	Yes (*71*)	Yes (may have originated millions of years ago) (*72*)
Lymphocytic Choriomeningitis Virus	Choriomeningitis	No (mainly in rodents, house mice, pet hamsters) (*73*)	Yes (*74*)	NA	30% (among infants diagnosed with congenital infection) (*74*)	Yes (*73*)	Yes (*75*)	Probably yes (LCMV is quite ancient and the extensive diversity of the virus has accumulated over the past 1,000–5,000 years) (*76*)
Aspergillus spp.	Aspergillosis	Yes (*77*)	No (most frequent cause of abortions in cattle) (*77,78*)	NA	86% (treated immunocompromis ed patients) (*77*)	Yes (*77*)	Yes (*79*)	No (first introduced in 1729) (*80*)

*NA, not available; SARS, severe acute respiratory syndrome; LCMV, lymphocytic choriomeningitis virus; HEV, hepatitis E virus; BC, before Christ.

References

1. Stenseth NC, Atshabar BB, Begon M, Belmain SR, Bertherat E, Carniel E, et al. Plague: past, present, and future. PLoS Med. 2008;5:e3. PubMed doi:10.1371/journal.pmed.0050003

2. Perry RD, Fetherston JD. *Yersinia pestis*—etiologic agent of plague. Clin Microbiol Rev. 1997;10:35–66. PubMed

3. Freemon FR. Bubonic plague in the Book of Samuel. J R Soc Med. 2005;98:436. PubMed doi:10.1258/jrsm.98.9.436

4. Bern C, Courtenay O, Alvar J. Of cattle, sand flies and men: a systematic review of risk factor analyses for South Asian visceral leishmaniasis and implications for elimination. PLoS Negl Trop Dis. 2010;4:e599. PubMed doi:10.1371/journal.pntd.0000599

5. Pagliano P, Carannante N, Rossi M, Gramiccia M, Gradoni L, Faella FS, et al. Visceral leishmaniasis in pregnancy: a case series and a systematic review of the literature. J Antimicrob Chemother. 2005;55:229–33. PubMed doi:10.1093/jac/dkh538

6. Seaman J, Mercer AJ, Sondorp E. The epidemic of visceral leishmaniasis in western Upper Nile, southern Sudan: course and impact from 1984 to 1994. Int J Epidemiol. 1996;25:862–71. PubMed doi:10.1093/ije/25.4.862

7. Roberts LJ, Handman E, Foote SJ. Science, medicine, and the future: Leishmaniasis. BMJ. 2000;321:801–4. PubMed doi:10.1136/bmj.321.7264.801

8. Cox FE. History of human parasitology. Clin Microbiol Rev. 2002;15:595–612. PubMed doi:10.1128/CMR.15.4.595-612.2002

9. Levett PN. Leptospirosis. Clin Microbiol Rev. 2001;14:296–326. PubMed doi:10.1128/CMR.14.2.296-326.2001

10. Vijayachari P, Sugunan AP, Shriram AN. Leptospirosis: an emerging global public health problem. J Biosci. 2008;33:557–69. PubMed doi:10.1007/s12038-008-0074-z

11. Hankins DG, Rosekrans JA. Overview, prevention, and treatment of rabies. Mayo Clin Proc. 2004;79:671–6. PubMed doi:10.4065/79.5.671

12. Windiyaningsih C, Wilde H, Meslin FX, Suroso T, Widarso HS. The rabies epidemic on Flores Island, Indonesia (1998-2003). J Med Assoc Thai. 2004;87:1389–93. PubMed

13. Labrique AB, Thomas DL, Stoszek SK, Nelson KE. Hepatitis E: an emerging infectious disease. Epidemiol Rev. 1999;21:162–79. PubMed

14. Navaneethan U, Al Mohajer M, Shata MT. Hepatitis E and pregnancy: understanding the pathogenesis. Liver Int. 2008;28:1190–9. PubMed doi:10.1111/j.1478-3231.2008.01840.x

15. Hosseini-Moghaddam SM, Zarei A, Alavian SM, Mansouri M. Hepatitis E virus infection: a general review with a focus on hemodialysis and kidney transplant patients. Am J Nephrol. 2010;31:398–407. PubMed doi:10.1159/000294505

16. Holmes KV. SARS coronavirus: a new challenge for prevention and therapy. J Clin Invest. 2003;111:1605–9. PubMed

17. Anderson RM, Fraser C, Ghani AC, Donnelly CA, Riley S, Ferguson NM, et al. Epidemiology, transmission dynamics and control of SARS: the 2002-2003 epidemic. Philos Trans R Soc Lond B Biol Sci. 2004;359:1091–105. PubMed doi:10.1098/rstb.2004.1490

18. Kahn JS, McIntosh K. History and recent advances in coronavirus discovery. Pediatr Infect Dis J. 2005 Nov;24(11 Suppl):S223-7, discussion S6.

19. Dos Reis M, Tamuri AU, Hay AJ, Goldstein RA. Charting the host adaptation of influenza viruses. Mol Biol Evol. 2010 Nov 25.

20. MacKenzie JS, Houghton M. Influenza infections during pregnancy: association with congenital malformations and with subsequent neoplasms in children, and potential hazards of live virus vaccines. Bacteriol Rev. 1974;38:356–70. PubMed

21. Mamelund SE. Effects of the Spanish Influenza pandemic on fertility and nuptiality in Norway. XXIV Conference on General Population; 2001 18-24 August; Salvador, Brazil; 2001.

22. Potter CW. A history of influenza. J Appl Microbiol. 2001;91:572–9. PubMed doi:10.1046/j.1365-2672.2001.01492.x

23. Guerra F. The earliest American epidemic. The influenza of 1493. Soc Sci Hist. 1988;12:305–25. PubMed doi:10.2307/1171451

24. Tellier R. Review of aerosol transmission of influenza A virus. Emerg Infect Dis. 2006;12:1657–62. PubMed

25. Martin PM, Martin-Granel E. 2,500-year evolution of the term epidemic. Emerg Infect Dis. 2006;12:976–80. PubMed

26. Sampathkumar P. West Nile virus: epidemiology, clinical presentation, diagnosis, and prevention. Mayo Clin Proc. 2003;78:1137–43, quiz 44. PubMed doi:10.4065/78.9.1137

27. Hayes EB, Komar N, Nasci RS, Montgomery SP, O'Leary DR, Campbell GL. Epidemiology and transmission dynamics of West Nile virus disease. Emerg Infect Dis. 2005;11:1167–73. PubMed

28. From the Centers for Disease Control and Prevention. Laboratory-acquired West Nile virus infections—United States, 2002. JAMA. 2003;289:414–5. PubMed doi:10.1001/jama.289.4.414

29. Galli M, Bernini F, Zehender G. Alexander the Great and West Nile virus encephalitis. Emerg Infect Dis. 2004;10:1330–2, author reply 2–3. PubMed

30. Gubler DJ. Dengue and dengue hemorrhagic fever. Clin Microbiol Rev. 1998;11:480–96. PubMed

31. Nandi S, Negi BS. Bovine ephemeral fever: a review. Comp Immunol Microbiol Infect Dis. 1999;22:81–91. PubMed doi:10.1016/S0147-9571(98)00027-7

32. Carroll ID, Toovey S, Van Gompel A. Dengue fever and pregnancy—a review and comment. Travel Med Infect Dis. 2007;5:183–8. PubMed doi:10.1016/j.tmaid.2006.11.002

33. Malavige GN, Fernando S, Fernando DJ, Seneviratne SL. Dengue viral infections. Postgrad Med J. 2004;80:588–601. PubMed doi:10.1136/pgmj.2004.019638

34. Pappas G, Akritidis N, Bosilkovski M, Tsianos E. Brucellosis. N Engl J Med. 2005;352:2325–36. PubMed doi:10.1056/NEJMra050570

35. Khan MY, Mah MW, Memish ZA. Brucellosis in pregnant women. Clin Infect Dis. 2001;32:1172–7. PubMed doi:10.1086/319758

36. Olsen S, Tatum F. Bovine brucellosis. Vet Clin North Am Food Anim Pract. 2010;26:15–27. PubMed doi:10.1016/j.cvfa.2009.10.006

37. Langmuir AD, Worthen TD, Solomon J, Ray CG, Petersen E. The Thucydides syndrome. A new hypothesis for the cause of the plague of Athens. N Engl J Med. 1985;313:1027–30. PubMed doi:10.1056/NEJM198510173131618

38. Ergonul O, Celikbas A, Tezeren D, Guvener E, Dokuzoguz B. Analysis of risk factors for laboratory-acquired brucella infections. J Hosp Infect. 2004;56:223–7. PubMed doi:10.1016/j.jhin.2003.12.020

39. Markovic-Denic L, Trifunovic VS, Zugic V, Radojic D, Stevanovic G. The first outbreak of brucellosis in the region of Sabac. Vojnosanit Pregl. 2010;67:634–7. PubMed doi:10.2298/VSP1008634M

40. Kyebambe PS. Acute brucella meningomyeloencephalo-spondylosis in a teenage male. Afr Health Sci. 2005;5:69–72. PubMed

41. Troutt HF, Osburn BI. Meat from dairy cows: possible microbiological hazards and risks. Rev Sci Tech. 1997;16:405–14. PubMed

42. Silver HM. Listeriosis during pregnancy. Obstet Gynecol Surv. 1998;53:737–40. PubMed doi:10.1097/00006254-199812000-00004

43. Gomez-Mampaso E, Baquero Mochales F, de Rafael Nerpel L, Michaux Oquinena L, Garcia-Villalba A. [Listeriosis and fertility (author's transl)]. Reproduccion. 1980;4:309–14. PubMed

44. Seeliger HP. Listeriosis—history and actual developments. Infection. 1988;16(Suppl 2):S80–4. PubMed doi:10.1007/BF01639726

45. Baum SG. Zoonoses—with friends like this, who needs enemies? Trans Am Clin Climatol Assoc. 2008;119:39-51; discussion -2.

46. Constantin CM, Martinelli AM, Foster SO, Bonney EA, Strickland OL. Smallpox: a disease of the past? Consideration for midwives. J Midwifery Womens Health. 2003;48:258–67, 302–4. PubMed doi:10.1016/S1526-9523(03)00084-9

47. Phadke AM, Samant NR, Dewal SD. Smallpox as an etiologic factor in male infertility. Fertil Steril. 1973;24:802–4. PubMed

48. Breman JG, Henderson DA. Diagnosis and management of smallpox. N Engl J Med. 2002;346:1300–8. PubMed doi:10.1056/NEJMra020025

49. Li Y, Carroll DS, Gardner SN, Walsh MC, Vitalis EA, Damon IK. On the origin of smallpox: correlating variola phylogenics with historical smallpox records. Proc Natl Acad Sci U S A. 2007;104:15787–92. PubMed doi:10.1073/pnas.0609268104

50. Coulibaly ND, Yameogo KR. Prevalence and control of zoonotic diseases: collaboration between public health workers and veterinarians in Burkina Faso. Acta Trop. 2000;76:53–7. PubMed doi:10.1016/S0001-706X(00)00090-5

51. Stein SJ, Greenspoon JS. Rubeola during pregnancy. Obstet Gynecol. 1991;78:925–9. PubMed

52. Elliman D, Sengupta N, El Bashir H, Bedford H. Measles, mumps, and rubella: prevention. Clin Evid (Online). 2007;2007.

53. Siegfried N, Wiysonge CS, Pienaar D. Too little, too late: measles epidemic in South Africa. Lancet. 2010;376:160. PubMed doi:10.1016/S0140-6736(10)61100-2

54. Furuse Y, Suzuki A, Oshitani H. Origin of measles virus: divergence from rinderpest virus between the 11th and 12th centuries. Virol J. 2010;7:52. PubMed doi:10.1186/1743-422X-7-52

55. Gorbach SL, Bartlett JG, Blacklow NR. Infectious Diseases. 3rd ed. Philadelphia: Lippincott Williams & Wilkins; 2004.

56. Awadalla SG, Mercer LJ, Brown LG. Pregnancy complicated by intraamniotic infection by Salmonella typhi. Obstet Gynecol. 1985;65(Suppl):30S–1S. PubMed

57. Crump JA, Luby SP, Mintz ED. The global burden of typhoid fever. Bull World Health Organ. 2004;82:346–53. PubMed

58. Meltzer E, Schwartz E. Enteric fever: a travel medicine oriented view. Curr Opin Infect Dis. 2010;23:432–7. PubMed doi:10.1097/QCO.0b013e32833c7ca1

59. Cabello F, Springer AD. Typhoid fever in Chile 1977-1990: an emergent disease [in Spanish]. Rev Med Chil. 1997;125:474–82. PubMed

60. Papagrigorakis MJ, Yapijakis C, Synodinos PN, Baziotopoulou-Valavani E. DNA examination of ancient dental pulp incriminates typhoid fever as a probable cause of the Plague of Athens. Int J Infect Dis. 2006;10:206–14. PubMed doi:10.1016/j.ijid.2005.09.001

61. Geiger JC. Tularemia in cattle and sheep. Cal West Med. 1931;34:154–6. PubMed

62. O'Toole D, Williams ES, Woods LW, Mills K, Boerger-Fields A, Montgomery DL, et al. Tularemia in range sheep: an overlooked syndrome? J Vet Diagn Invest. 2008;20:508–13. PubMed doi:10.1177/104063870802000417

63. Feldman KA, Enscore RE, Lathrop SL, Matyas BT, McGuill M, Schriefer ME, et al. An outbreak of primary pneumonic tularemia on Martha's Vineyard. N Engl J Med. 2001;345:1601–6. PubMed doi:10.1056/NEJMoa011374

64. Trevisanato SI. The biblical plague of the Philistines now has a name, tularemia. Med Hypotheses. 2007;69:1144–6. PubMed doi:10.1016/j.mehy.2007.02.036

65. Fredriksson-Ahomaa M, Stolle A, Korkeala H. Molecular epidemiology of *Yersinia enterocolitica* infections. FEMS Immunol Med Microbiol. 2006;47:315–29. PubMed doi:10.1111/j.1574-695X.2006.00095.x

66. Platt-Samoraj A, Szweda W, Procajlo Z. The influence of experimental *Yersinia enterocolitica* infection on the pregnancy course in sows—preliminary studies. I. Bacteriological examination. Pol J Vet Sci. 2009;12:317–22. PubMed

67. Vento S, Cainelli F, Cesario F. Infections and thalassaemia. Lancet Infect Dis. 2006;6:226–33. PubMed doi:10.1016/S1473-3099(06)70437-6

68. Gourdon F, Beytout J, Reynaud A, Romaszko JP, Perre D, Theodore P, et al. Human and animal epidemic of *Yersinia enterocolitica* O:9, 1989–1997, Auvergne, France. Emerg Infect Dis. 1999;5:719–21. PubMed doi:10.3201/eid0505.990516

69. Bottone EJ. *Yersinia enterocolitica*: the charisma continues. Clin Microbiol Rev. 1997;10:257–76. PubMed

70. Bechah Y, Capo C, Mege JL, Raoult D. Epidemic typhus. Lancet Infect Dis. 2008;8:417–26. PubMed doi:10.1016/S1473-3099(08)70150-6

71. Raoult D, Ndihokubwayo JB, Tissot-Dupont H, Roux V, Faugere B, Abegbinni R, et al. Outbreak of epidemic typhus associated with trench fever in Burundi. Lancet. 1998;352:353–8. PubMed doi:10.1016/S0140-6736(97)12433-3

72. Andersson SG, Zomorodipour A, Andersson JO, Sicheritz-Ponten T, Alsmark UC, Podowski RM, et al. The genome sequence of *Rickettsia prowazekii* and the origin of mitochondria. Nature. 1998;396:133–40. PubMed doi:10.1038/24094

73. Fischer SA, Graham MB, Kuehnert MJ, Kotton CN, Srinivasan A, Marty FM, et al. Transmission of lymphocytic choriomeningitis virus by organ transplantation. N Engl J Med. 2006;354:2235–49. PubMed doi:10.1056/NEJMoa053240

74. Jamieson DJ, Kourtis AP, Bell M, Rasmussen SA. Lymphocytic choriomeningitis virus: an emerging obstetric pathogen? Am J Obstet Gynecol. 2006;194:1532–6. PubMed doi:10.1016/j.ajog.2005.11.040

75. Baum SG, Lewis AM Jr, Rowe WP, Huebner RJ. Epidemic nonmeningitic lymphocytic-choriomeningitis-virus infection. An outbreak in a population of laboratory personnel. N Engl J Med. 1966;274:934–6. PubMed doi:10.1056/NEJM196604282741704

76. Albarino CG, Palacios G, Khristova ML, Erickson BR, Carroll SA, Comer JA, et al. High diversity and ancient common ancestry of lymphocytic choriomeningitis virus. Emerg Infect Dis. 2010;16:1093–100. PubMed doi:10.3201/eid1607.091902

77. Latge JP. *Aspergillus fumigatus* and aspergillosis. Clin Microbiol Rev. 1999;12:310–50. PubMed

78. McCausland IP, Slee KJ, Hirst FS. Mycotic abortion in cattle. Aust Vet J. 1987;64:129–32. PubMed doi:10.1111/j.1751-0813.1987.tb09659.x

79. Buffington J, Reporter R, Lasker BA, McNeil MM, Lanson JM, Ross LA, et al. Investigation of an epidemic of invasive aspergillosis: utility of molecular typing with the use of random amplified polymorphic DNA probes. Pediatr Infect Dis J. 1994;13:386–93. PubMed doi:10.1097/00006454-199405000-00011

80. Andrade-Filho Jde S, Pena GP. Analogies in medicine: fungus and liturgy. Rev Inst Med Trop Sao Paulo. 2010;52:288. PubMed doi:10.1590/S0036-46652010000500014
